# *In vitro* Cytokine Responses to Virulent PRRS Virus Strains

**DOI:** 10.3389/fvets.2020.00335

**Published:** 2020-07-15

**Authors:** Gianluca Ferlazzo, Jessica Ruggeri, Maria Beatrice Boniotti, Flavia Guarneri, Ilaria Barbieri, Matteo Tonni, Cristina Bertasio, Giovanni Loris Alborali, Massimo Amadori

**Affiliations:** ^1^Laboratory of Animal Welfare, Clinical Chemistry and Veterinary Immunology, Istituto Zooprofilattico Sperimentale della Lombardia e dell'Emilia Romagna, Brescia, Italy; ^2^Genomics Department, Istituto Zooprofilattico Sperimentale della Lombardia e dell'Emilia Romagna, Brescia, Italy; ^3^Diagnostic Laboratory, Istituto Zooprofilattico Sperimentale della Lombardia e dell'Emilia Romagna, Brescia, Italy

**Keywords:** pig, PRRS, virus, leukocytes, inflammatory response, pathogenicity

## Abstract

Porcine reproductive and respiratory syndrome (PRRS) affects farmed swine causing heavy direct and indirect losses. The infections sustained by PRRS viruses (PRRSV-1 and PRRSV-2) may give rise to severe clinical cases. This highlights the issue of PRRSV pathogenicity and relevant markers thereof. Since PRRSV strains can be discriminated in terms of immunotypes, we aimed to detect possible correlates of virulence *in vitro* based on the profile of innate immune responses induced by strains of diverse virulence. To this purpose, 10 field PRRSV isolates were investigated in assays of innate immune response to detect possible features associated with virulence. Tumor necrosis factor-α, interleukin (IL)-1beta, IL-8, IL-10, and caspase-1 were measured in cultures of PRRSV-treated peripheral blood mononuclear cells of PRRS-naive pigs, unable to support PRRSV replication. Two reference PRRSV strains (highly pathogenic and attenuated, respectively), were included in the screening. The PRRSV strains isolated from field cases were shown to vary widely in terms of inflammatory cytokine responses *in vitro*, which were substantially lacking with some strains including the reference, highly pathogenic one. In particular, neither the field PRRSV isolates nor the reference highly pathogenic strain gave rise to an IL-1beta response, which was consistently induced by the attenuated strain, only. This pattern of response was reversed in an inflammatory environment, in which the attenuated strain reduced the ongoing IL-1beta response. Results indicate that some pathogenic PRRSV strains can prevent a primary inflammatory response of PBMCs, associated with reduced permissiveness of mature macrophages for PRRSV replication in later phases.

## Introduction

Porcine reproductive and respiratory syndrome (PRRS) is sustained by two distinct Arteriviruses, named PRRSV-1 and PRRSV-2 (1). This is one of the most important swine diseases worldwide, with estimated losses at $664 million every year in the USA and a trend to increasing prevalence in the last years (2, 3). A similar situation is found in Europe, even though the European PRRSV-1 strains differ from the American PRRSV-2 ones in terms of virulence pattern and major biological properties (4). Importantly, PRRSV infections are often characterized by reduced and delayed adaptive immune responses in terms of neutralizing antibody and PRRSV-specific interferon-gamma (IFN-gamma) secreting cells, as markers of virus-specific, cell-mediated immunity (5). In addition, no virus-specific immune responses have been convincingly correlated with clinical and virological protection in PRRSV-infected pigs. In particular, the time course of viremia is substantially unrelated to the time course of the adaptive immune response (5). Thus, immune sera containing neutralizing antibody can confer passive clinical protection against a subsequent challenge in sows, but the treatment does not reduce the viral load in tissues (6), and no protecting effects are observed in pigs after PRRSV infection, in which viremia and neutralizing antibody may coexist for several weeks (7). These data are in agreement with the murine Arterivirus model, in which the infection shows the same time course in both immunotolerant and control mice (8). Finally, the clinical features observed experimentally in isolation facilities are often quite different from those found in field cases (9), which points at a major role of environmental, infectious, and non-infectious stressors in the onset of full-blown clinical disease (10). The same discrepancy between field and isolation facilities also refers to inactivated and attenuated PRRS vaccines, the impact of which may be sometimes worse than expected on farm (11).

Recent studies showed that PRRSV strains can be grouped in immunotypes, [i.e., types differing in their interaction with the innate immune system *in vitro* (12)]. Thus, PRRSV strains induce distinct profiles of innate immune response in PBMCs (12), which show little, if any, competence for PRRSV replication (13). In this respect, the results obtained on PBMCs of PRRS-naive pigs point at fundamental properties of PRRSV in the regulation of the innate immune response without the confounding effects of virus replication and previous, virus-specific, adaptive immune responses. Immunotypes of PRRSV probably underlie differences of clinical and epidemiological impact, as described in previous studies matching clinical forms and *in vivo* immune responses (14). Therefore, our working hypothesis implied that crucial, virus-driven events related to the innate immune response might take place before the differentiation of mature macrophages and bear on the host's susceptibility to PRRSV infection. This was the fundamental background of our study. Accordingly, our main objective was to define profiles of innate immune response *in vitro* discriminating between virulent and non-virulent PRRSV strains. In particular, our methodology implied the detection and the measurement of inflammatory cytokine responses *in vitro* under precise, standardized conditions, using leukocytes of different PRRS-naive pigs to cope with individual variability of the innate immune response. For a correct interpretation of test results, two reference, highly pathogenic, and attenuated PRRSV strains, respectively, were introduced into a panel of PRRSV isolates from disease cases to be tested *in vitro*.

## Materials and Methods

### Animals

This study complied with Italian laws on animal experimentation and ethics. The clinical trial on PRRSV strain BS773 was carried out within a specific animal experiment license issued by the Italian Ministry of Health (n. 631/2017-PR of 7 August 2017). Swine PBMCs of PRRS-naive, SPF pigs had been obtained in previous studies and stored in liquid nitrogen. PRRSV strains had been isolated from field cases in the framework of our PRRS surveillance activity.

### Macrophages and PRRSV Strains

Heparinized blood of healthy, PRRSV antibody-negative, SPF pigs was diluted 1:2 with Roswell Park Memorial Institute (RPMI) 1640 medium, and PBMCs were separated by centrifugation on Histopaque® 1.077 (code 10771, Merck KGaA, Darmstadt, Germany) at 1,100 *g*, 25 min, 20°C, and stored in liquid nitrogen at 5 × 10^6^/ml in RPMI 1640 medium + 40% FCS and 10% dimethyl sulfoxide (DMSO). Then, PBMCs were thawed and grown at 6 × 10^6^/ml in RPMI 1640 medium at 37°C in 5% CO_2_ in microtiter plates over 2.5 h. Non-adherent cells were discarded, and adherent monocytes were further cultivated in RPMI 1640 medium supplemented with 10% FCS and 10 ng/ml human macrophage Colony Stimulating Factor (CSF) (code M6518-10UG, Sigma-Aldrich) over 4 days.

PAM are routinely obtained in our laboratory by bronchoalveolar lavage of healthy, SPF, PRRS-naive pigs as previously described (15), on the basis of a permanent animal license. PAM of each animal are separately processed and stored in aliquots in liquid nitrogen. They constitute a single batch deriving from one pig only, which is duly checked for susceptibility to different PRRSV strains. After passing the tests, the cells of this batch are deemed representative of PRRSV-susceptible animals without the disturbing effects of environmental microbial pathogens, often observed in non-SPF animals. A single batch of highly sensitive and replication-competent PAM was selected only for virus propagation over the whole study to minimize the variability of test results. Cells were thawed on the day before the experiment and grown in microtiter plates at 10^6^/ml in RPMI 1640 medium supplemented with 20% FCS at 37°C in 5% CO_2_. These cells are not adherent to plastic and can be easily recovered by gently pipetting. More than 90% viability was always detected after thawing the frozen vials.

Twenty PRRSV strains were obtained from field cases of either respiratory or reproductive disease (Table 1). The presence of PRRSV was confirmed by real-time, reverse transcription (RT)-PCR, as described in the following section. Open reading frame (ORF) five and seven genes of some strains were sequenced, and the relevant gene bank accession numbers are reported in Table S1.

**Table 1:** PRRSV strains included in the study.

**Sample registration number/PRRSV strains**	**Primary diagnosis, date**	**Specimen**	**Clinical form on farm**
270433/2 and 5	12.2016	Blood	Reproductive
271009/6 and 8	12.2016	Blood	Respiratory
3400/2	09.01.2017	Blood	Reproductive
13957	25.01.2017	Lung homogenate	Respiratory and reproductive
21377	25.01.2017	Lung homogenate	Respiratory and reproductive
24663/1, 2, and 4	26.01.2017	Blood	Reproductive
127669/1, 6, 8, 9, and 10	10.05.2017	Fetal lung homogenate	Reproductive
148903	30.05.2017	Fetal lung homogenate	Reproductive
150631/1, 2, 3, and 5	31.06.2017	Fetal lung homogenate	Reproductive

*The diagnostic samples included in the study are reported in the first column on the left. If the consignment consisted of one sample only, the relevant PRRSV strain was named after the corresponding registration number. If the consignment consisted of a few samples, each isolated PRRSV strain was named after the corresponding registration number followed (/) by the sample number. PRRSV strains are sometimes reported in a shortened form for convenience*.

The attenuation of PRRSV strain BS114 had been previously reported (16). The BS773 strain had been isolated in PAM from a PRRS outbreak in weaners, characterized by a severe respiratory syndrome with very high morbidity and mortality.

### Real-Time RT-PCR for PRRSV ORF 7

Viral RNA was extracted from 200 μl of sample (serum or lung homogenate) using a commercial kit (NucleoMag® Vet kit, Macherey-Nagel, Düren, Germany), according to the manufacturer's instructions. An exogenous internal control RNA (Xeno^TM^ RNA control, Applied Biosystem) was added to specimens prior to RNA extraction to verify the success of the procedure and the absence of inhibitors. The extraction was carried out on the Biosprint 96 instrument (Qiagen, Hilden, Germany), using the NucleoMag Vet 200 protocol. Nucleic acids were eluted into 100 μl of elution buffer and immediately subjected to RT-PCR or stored at −80°C until used. RNA was detected using the LSI VetMAX^(TM)^ PRRSV EU/NA Real-Time PCR kit (Applied Biosystem), as specified by the manufacturer.

Ten-fold serial dilutions in nuclease-free water of an EU PRRSV RNA (3.5 × 10^5^ to 35 copies/μl) were used to generate a standard curve and to quantify PRRSV RNA in the samples. Triplicates of each dilution were run in each assay. The following equation: $\text{x}=10\frac{\text{Cq}-\left(\text{yintercept}\right)}{\text{slope}},$

where *x* represents the genome copies per microliter and was used to transform the *Cq* values of the samples into estimates of genome copies of PRRSV RNA per milliliter.

The detection limit of PRRSV-1 target was nine copies of nucleic acids per reaction, corresponding to 0.642 genome copies/μl.

### Isolation and Propagation of PRRSV Strains

PRRSV strains from field cases (Table 1) were diluted 1:10 in RPMI 1640 medium + 2% FCS and filtered through 0.2 μm membranes. Next, 0.2 ml of each virus strain was adsorbed onto 4-day old adherent macrophages from PBMCs in six-well microtiter plates. After 2 h at 37°C, 5% CO_2_, the inoculum was discarded, and cells were washed twice with RPMI 1640 medium. Next, 4.5 ml of RPMI 1640 medium + 2% FCS was added to each well, and plates were again incubated at 37°C, 5% CO_2_ for 1 h. Then, 0.5-ml supernatant samples were collected from each well and stored at −80°C (day 0 samples). Plates were again incubated at 37°C, 5% CO_2_ and visually examined for CPE over 2–3 days. Next, plates were set at −80°C for 2 h at least before thawing and collecting again 0.5-ml samples from each well. Amplification of PRRSV strains was evaluated on the basis of CPE and quantified by real-time RT-PCR. The same procedure was also applied to 1-day old PAM cultures in microtiter plates. In this case, cells were washed by centrifugation (330 *g*, 5 min, 20°C) after virus absorption.

### Clinical Trial

The pathogenicity of PRRSV strain BS773 was checked in a clinical trial in isolation facilities. Two groups of 6, 3-month old pigs were selected for the study. They were obtained from a seronegative farm with no evidence of PRRSV infection. Group 1 was inoculated intranasally with 10^6^ TCID_50_ of PRRSV strain BS773, whereas group 2 received sterile saline and represented the negative control of the study. All the animals were submitted to clinical inspections on a daily basis over 2 weeks. Venous blood samples were collected to evaluate viremia and to monitor cytokine responses to PRRSV infection.

### Incubation of Swine PBMCs With PRRSV

PBMCs from SPF, PRRS-naive pigs were thawed at 38°C, washed twice in RPMI 1640 medium (330 *g*, 10 min, 20°C) and resuspended at 10^7^/ml in RPMI 1640 medium + antibiotics (penicillin, 50 U/ml; streptomycin, 50 μm/ml; amphotericin B, 2 μm/ml; Thermo Fisher Scientific, catalog numbers 15140122 and 11805017), and 10% FCS. Next, 0.25 ml/well (i.e., 2.5 × 10^6^ cells) was seeded into 48-well tissue culture plates and incubated at 37°C in 5% CO_2_. PBMCs were reacted with 0.25 ml/well of medium (control) and the PRRSV strains under study, respectively, at MOI 2, (i.e., 5 × 10^6^ genome copies/well). Plates were incubated at 37°C in 5% CO_2_ for 18 h and centrifuged at 425 *g*, 10 min, 5°C. The supernatant of each well was collected and stored in aliquots at −80°C, whereas the plate with pelleted cells was stored at 4°C for the colorimetric caspase-1 assay, performed within the same working session. For each PRRSV strain under study, this procedure was repeated with PBMCs of at least four PRRS-naive pigs.

### Flow Cytometry Analyses

PAM were collected from 1-day old cultures by gentle pipetting. Plastic-adherent macrophages from blood monocytes were washed twice with Ca- and Mg-free phosphate-buffered saline (PBS) and detached in ethylenediaminetetraacetic acid (EDTA) 10 mM in PBS over 1 h at 4°C, followed by gentle scraping with the tip of a micropipette. After washing twice in FCB (i.e., PBS + 2% heat-inactivated FCS + 0.1% azide), cells were resuspended at 6 × 10^6^/ml in FCB. Fifty microliters/well of this suspension were distributed in four wells of a 96-well, U-bottomed microtiter plate. In order to check the suitability of our cells for high-titered replication of PRRSV, cells of two wells were reacted with 25 μl of pretitered monoclonal antibodies 2A10/11 to porcine CD163 (Bio-Rad) and 3B11/11 to porcine CD169 (Bio-Rad), respectively, involved in binding and internalization of PRRSV (17). Two wells were reacted with FCB only (control). After 30 min at 4°C, 100 μl/well of FCB were added, and plates were centrifuged at 500 *g*, 3 min, 4°C. The plates were flicked over a sink, and all wells but one (cell control) were reacted with 25 μl/well of Alexa Fluor® 488 F(ab′)2 fragment of goat antimouse immunoglobulin G (IgG), IgM (H + L) (Thermo Fisher, catalog number A10684). After 30 min at 4°C, cells were washed twice (500 *g*, 3 min, 4°C) with 100 μl/well FCB and resuspended in 200 μl/well FCB. Two microliters/well of propidium iodide (PI) at 50 μg/ml (BD Pharmingen, catalog number 51-66211E) were added, and cells were analyzed in a Guava EasyCyte HT flow cytometer using Incyte software. Green fluorescence of PI-negative (viable) cells was evaluated with respect to the secondary antibody control, whereas forward and side scatter signals were calibrated on the basis of the unreacted control cells.

Active caspase-1 was detected in PBMC cultures by the FAM-FLICA flow cytometry assay, using a commercial kit as specified by the manufacturer (FAM-FLICA Caspase Assay, catalog number 98, Immunochemistry Technologies, Bloomington, MN, USA). This reveals active caspases 1, 4, and 5 by means of a fluorochrome-tagged YVAD probe. To this purpose, PBMCs of PRRS-naive pigs were stimulated with different PRRSV strains as described above in agarose-treated, 48-well microtiter plates, to prevent the adhesion of monocytes; a positive control of the inflammasome reaction was set up with a combination of LPS (1 μg/ml) and ATP (4.9 mM) treatments, as previously described (18). Baseline conditions were defined in untreated PBMCs stained with the FLICA reagent. Late apoptotic and necrotic cells were discriminated by means of PI treatment just before the analysis.

### Porcine Cytokine Assays

Tumor necrosis factor alpha (TNF-α) was determined by a cytotoxicity assay on actinomycin D-treated WEHI 164 cells (19). Cytokine concentrations were determined from a standard curve created with a reference preparation of swine recombinant TNF-α (Pierce Endogen, Rockford, USA).

IL-1beta was measured by a commercial kit (porcine IL-1 beta/IL-1F2 DuoSet ELISA, R&D Systems, catalog number DY681), as specified by the manufacturer.

IL-10 was measured by a commercial kit (porcine IL-10 DuoSet ELISA, R&D Systems, catalog number DY693B) according to the manufacturer's directions.

IL-8 was measured by a commercial ELISA kit (R&D systems, DUOset, catalog number DY535).

The above assays were carried out on PRRSV-treated and control PBMCs cultures.

*In vivo*, sera of PRRSV-infected and control pigs were checked for interferon-gamma by sandwich ELISA with monoclonal antibodies P2F6 (capture) and biotinylated MP701B (tracer) (Thermo Scientific, Rockford, IL, USA), as previously described (11).

### Colorimetric Caspase-1 Assay

This was carried out on pelleted PBMCs after incubation with medium (control) and PRRSV strains, respectively, using caspase-1/ICE Colorimetric Assay Kit (BioVision, catalog number K111-200) as specified by the manufacturer, with minor changes and under strict cold chain conditions. Briefly, 50 μl/well of the kit's cell lysis buffer was added to the cell pellet, and plates were incubated for 10 min over crushed ice. Plates were again centrifuged at 2,200 *g*, 10 min, 4°C. Each supernatant was transferred to a well of an ELISA plate (NUNC Maxisorp flat-bottom, catalog number 44-2404-21, Thermo Fisher Scientific) and reacted with 50 μl/well of the kit reaction buffer. This was also added to the positive control (1 μl caspase-1 standard + 49 μl of reaction buffer) and to the negative control (50 μl/well of cell lysis buffer). Then, 5 μl/well was added of chromogen YVAD-pNA. Plates were incubated at 37°C, 5% CO_2_ per 1.5 h and read spectrophotometrically at 405 nm. Results were expressed in terms of DeltaOD at 405 nm, (i.e., OD_405nm_ sample – OD_405nm_ negative control).

### Statistical Analyses

For each cytokine assay, the prevalence of test-positive and test-negative results in all the PBMC cultures was analyzed by a chi-square test. This was also used to check the prevalence of caspase-1-positive cells under different experimental conditions in the flow cytometry assay. The Fisher's exact test was adopted to analyze the prevalence of IFN-gamma-positive sera in PRRSV-infected and control pigs. The significance threshold was set at *P* < 0.05 (Graph Pad Prism 5, GraphPad Software Inc., La Jolla, CA, USA).

## Results

### Propagation of PRRSV Strains

**Figure 1:**
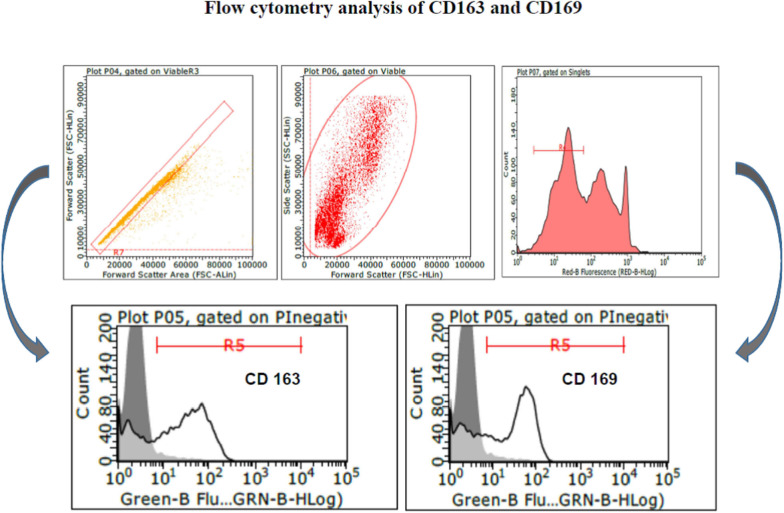
Swine PAM were stained with monoclonal antibodies 2A10/11 to porcine CD163 and 3B11/11 to porcine CD169, respectively, or FCB (control). Next, cells were reacted with Alexa Fluor® 488 F(ab′)_2_ fragment of goat antimouse IgG, IgM (H + L). After the final washing, cells were stained with PI and gated by a combination of forward and side scatter (FSC vs. SSC). Next, doublet and multiplet cells were discriminated from single cells in a FSC height/FSC area cytogram. Single, PI-negative (viable) cells were gated to histograms depicting the expression of CD163 and CD169, respectively. Dark area in the histograms: control reaction.

PAM and macrophage cells from PBMCs expressed CD163 and CD169 under the aforementioned conditions (Figure 1) and could sustain high-titered replication of all the PRRSV strains under study.

### Clinical Trial

Intranasal administration of 10^6^ TCID_50_ of PRRSV BS773 induced pyrexia, decreased alertness, anorexia, dyspnea, cough, and nasal discharge over 2 weeks after infection in 3-month old, PRRS-naive pigs (Table 2). All the PRRSV-infected animals showed viremia peaking at 7 DPI (mean, 1.05 × 10^7^; SD, 6.91 × 10^6^ genome copies/ml). In addition, PRRSV BS773 induced strong IFN-gamma responses peaking at 3 and 7 DPI. These clinical and cytokine responses were not observed in control, non-infected pigs (Table 2). On the basis of these results, BS773 was included in our study as reference, highly pathogenic PRRSV strain.

**Table 2A:** Clinical signs of BS773 PRRSV infection.

		**0 DPI**	**1 DPI**	**2 DPI**	**3 DPI**	**4 DPI**	**5 DPI**	**6 DPI**	**7 DPI**	**8 DPI**	**9 DPI**	**10 DPI**	**11 DPI**	**12 DPI**	**13 DPI**	**14 DPI**
Group 1 PRRSV	Fever^a^	0/6	0/6	6/6	5/6	5/6	0/6	6/6	5/6	6/6	6/6	5/6	5/6	4/6	0/6	0/6
	General condition score^b^	0/6	0/6	0/6	0/6	0/6	0/6	0/6	0/6	1/6	6/6	6/6	6/6	4/6	6/6	6/6
	Respiratory signs^c^	0/6	0/6	0/6	3/6	3/6	1/6	1/6	2/6	5/6	6/6	6/6	6/6	6/6	6/6	5/6
Group 2 control	Fever^a^	0/6	0/6	0/6	0/6	0/6	0/6	0/6	0/6	0/6	0/6	0/6	0/6	0/6	0/6	0/6
	General condition score^b^	0/6	0/6	0/6	0/6	0/6	0/6	0/6	0/6	0/6	0/6	0/6	0/6	0/6	0/6	0/6
	Respiratory signs^c^	0/6	0/6	0/6	0/6	0/6	0/6	0/6	0/6	0/6	0/6	0/6	0/6	0/6	0/6	0/6

*Figures of clinical signs are shown as disease-positive animals out of the total. From DPI 2 to 12 pigs inoculated intranasally with PRRSV BS773 strain (Group 1) showed pyrexia, while general condition worsened from DPI 8 onwards. Respiratory signs were evident from DPI 3 and still present at DPI 14. Control animals (Group 2) did not show pyrexia or clinically evident signs at any time point of the experiment*.

a *Rectal temperature ≥40°C was considered as fever*.

b *General condition score was defined based on appetite level: 0, normal; 1, decreased; 2, absent*.

c *Respiratory signs were also scored from 0 to 6 as follows: 0, normal; 1, mild dyspnea and/or tachypnea when stressed; 2, mild dyspnea and/or tachypnea at rest; 3, moderate dyspnea and/or tachypnea when stressed; 4, moderate dyspnea and/or tachypnea at rest; 5, severe dyspnea and/or tachypnea when stressed; 6, severe dyspnea and/or tachypnea at rest (20)*.

**Table 2B:** IFN-gamma response in serum samples.

	**PRRSV BS773**	**Controls**
DPI 0	1/6	0/6
DPI 3	4/6	0/6
DPI 5	2/6	0/6
DPI 7	5/6	1/6

*Results are shown as IFN-gamma-positive animals out of the total. The prevalence of cytokine responses was significantly higher in PRRSV-infected animals (P < 0.01 for the whole period under study)*.

### Cytokine Responses to PRRSV Strains

Ten PRRSV strains out of 20 reported in Table 1 were employed for the stimulation of four to six PBMC cultures of as many PRRS-naive pigs (Table 3); the 10 PRRSV strains corresponded to the highest levels of replication in macrophage cultures. Results were interpreted as shown in the legend to Table 3. Although PRRSV strains sometimes provided different test results in PBMC cultures of different pigs (*P* < 0.01 in IL-8 and IL-10 assays), an IL-8 response was induced by most PRRSV strains in agreement with previous results (12); other markers were expressed by fewer isolates. Importantly, PRRSV strains 21377 and 669/6 induced little if any inflammatory response in PBMCs cultures, as opposed to the other strains under study (Table 3). On the whole, four major patterns of response to PRRSV were evidenced, corresponding to PRRSV strains 433/5, 009/6, 009/8, 21377 (Table 2). As a caveat, it should be stressed that PRRSV strains were not tested on PBMCs cultures of the same pigs, which might affect PRRSV profiling.

**Table 3:** Assays of innate immunity in PBMCs cultures.

**PRRSV**	**Delta pg/ml of cytokine concentrations (PRRSV treated–control)**				**Delta mOD (PRRSV treated–control)**
	**IL-8**	**TNF-alpha**	**IL-10**	**IL-1beta**	**Caspase-1**
433/5	+ (8,371)	neg	neg	neg	+ (32)
009/6	+ (5,031)	neg	+ (219) *	neg	neg
009/8	+ (6,317)	+ (328)	neg	neg	neg
21377	+ (2,251)	neg	neg	neg	neg
433/2	+ (3,642)	+ (144)	+ (363) *	ND	neg
3400/2	neg	+ (34)	++ (127) *	ND	neg
13957	+ (320)	+ (236)	neg	ND	neg
669/1	+ (8,957)	+ (57)	neg	ND	+ (23)
669/6	+ (447)	neg	neg	ND	neg
631/1	+ (4,160)	neg	neg	ND	+ (14)
Ref. highly pathogenic BS773	+ (821)	neg	neg	neg	neg
Ref. attenuated BS114	+ (18,157)	neg	neg	++ (96)	neg

*Each PRRSV strain was tested on four to six PBMCs cultures from PRRS-naive pigs at MOI 2. For each PBMCs culture, PRRSV strains were scored test positive if IL-8, TNF-alpha, IL-1beta, and IL-10 release was increased at least two-fold with respect to untreated, control cultures. For the colorimetric caspase-1 assay, a 10-mOD increase was considered as threshold. PRRSV strains were scored weakly positive if the cytokine release was increased less than the above limits. PRRSV strains increasing cytokine release in at least half of the PBMCs cultures under study are reported as positive (+) in the table and the mean increase in cytokine concentration beyond the spontaneous release in control PBMCs cultures is reported between brackets in terms of pg/ml. PRRSV strains are reported as highly positive (++) in the table if the response was induced in all the PBMCs cultures under study. The presence of IL10-positive PRRSV strains is highlighted with an asterisk*.

*Neg, < 50% of test-positive results; ND, not done*.

Then, the four selected PRRSV strains were compared with a reference attenuated (BS114) and a reference, highly pathogenic (BS773) PRRSV strain, according to the results of our clinical trial. In order to obtain further information, the IL-1beta response was also taken into account. Results (Table 3) showed that only the attenuated BS114 strain gave rise to a strong IL-1beta response in PBMCs of PRRS-naive pigs. The highly pathogenic BS773strain induced no response in PBMC cultures, whereas strains 009/6, 433/2, and 3400/2 caused an IL-10 response, in agreement with previous data on PRRSV-1 (12). A huge difference between the two reference PRRSV strains was also demonstrated in terms of IL-8 response (see Table 3). On the whole, the clinical attenuation features of strain BS114 were correlated with the IL-1beta and IL-8 markers in PBMCs reacted with PRRSV under non-inflammatory conditions.

### IL-1Beta Response Under Inflammatory Conditions

**Figure 2:**
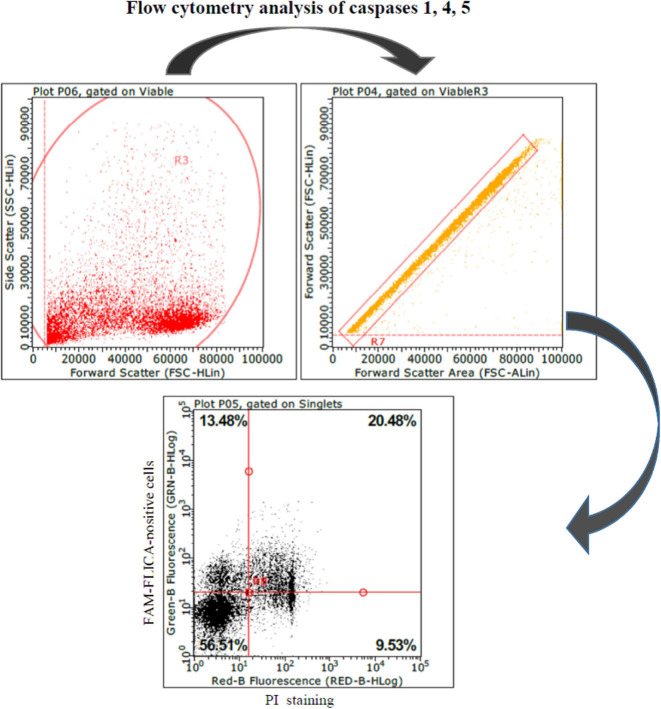
Swine PBMCs were stained with the FAM-FLICA YVAD probe. After the final washing, cells were stained with propidium iodide and gated by a combination of forward and side scatter (FSC vs. SSC). Next, doublet and multiplet cells were discriminated from single cells in a FSC height/FSC area cytogram. Single cells were gated to a quadrant cytogram, depicting caspase-positive cells in the *y*-axis, and dead (late apoptotic and necrotic) cells in the *x*-axis.

These results led us to investigate the possible effects of PRRSV strains under inflammatory conditions. We decided first to carry out a screening of spontaneous IL-1beta release by PBMC cultures of PRRS-naive pigs, and the least responsive batch was eventually selected. As a model of inflammatory conditions, we chose monocytes of PBMCs exposed to: (i) detachment-induced cell death (anoikis) (24) in agarose-treated microplates and (ii) LPS. In the FAM-FLICA flow cytometry assay (Figure 2), the attenuated BS114 strain caused, in the presence of LPS, the largest increase in the prevalence of live, caspase-1-positive cells, as well as a strict containment of apoptosis/necrosis; it also caused a strong reduction in the ongoing IL-1beta response (24), as opposed to the pathogenic PRRSV strains under study (see Table 4).

Table 4Caspase-1 and IL-1beta expression in PBMCs under inflammatory conditions.

**Table:** 

**(A) % live and dead, caspase 1-positive cells in flow cytometry**								
**PRRSV strains**	**Virus only**		**Virus** **+** **LPS**		**Delta %**		***P*****-values**	
	**%Live**	**%Dead**	**%Live**	**%Dead**	**Live**	**Dead**	**Live**	**Dead**
BS773	7.61	27.00	8.04	32.08	+0.43	+5.08	NS	<0.01
21377	8.47	27.28	8.84	31.59	+0.37	+4.31	NS	<0.01
433/5	6.12	25.43	6.07	28.65	−0.05	+3.22	NS	<0.01
BS114 attenuated	7.21	29.41	8.86	29.20	+1.65	−0.21	<0.01*	NS
**Controls**			**%Live**					**%Dead**
LPS only			7.72					34.76
LPS + ATP			9.39					19.07
Medium only			6.21					29.15

**Table:** 

**(B) IL-1beta release from PRRSV and LPS-treated PBMC cultures**			
**PRRSV strains**	**Virus only (pg/ml)**	**Virus** **+** **LPS (pg/ml)**	**Delta: (virus** **+** **LPS) – LPS only**
BS773	2,655	>8,000	≥674
21377	6,442	7,145	−181
433/5	6,195	>8,000	≥674
BS114	1,892	2,786	−4,540
**Controls**			**pg/ml**
LPS only			7,326
LPS + ATP			6,040
Medium only			4,315

*Aliquots of PBMCs of six PRRS-naive pigs in liquid nitrogen were thawed and screened for spontaneous IL-1beta release. Then, a further aliquot of the PBMCs showing the lowest level of release was thawed, and cells were grown in 0.5 ml/well of RPMI 1640 medium + 10% heat-inactivated FCS and LPS (1 μg/ml final) at 37°C, 5% CO_2_, in agarose-treated, 48-well microtiter plates to induce detachment-induced cell death (anoikis). After 4 h, different PRRSV strains at MOI 2 or ATP (4.9 mM final, inflammasome control) were added, and PBMCs were further grown for another 18 h. Then, 150 μl/well of supernatant were carefully removed to investigate the IL-1 beta response, whereas the cells were employed in a FAM-FLICA flow cytometry assay for caspase-1. Singlet cells were analyzed in a quadrant cytogram for green (caspase-1) and red (PI) fluorescence as shown in Figure 2. The two experiments were carried out on four PRRSV strains. **(A)** % live, caspase 1-positive cells and the corresponding dead ones (late apoptotic and necrotic) are reported. For each result, a delta value is reported as the % difference between (virus + LPS) and (virus only). For each delta value, the corresponding P value is shown in the next column on the right. NS, not significant. The significant increase of live, caspase 1-positive cells in attenuated PRRSV-stimulated PBMCs is highlighted with an asterisk. **(B)** The concentrations of IL-1beta in the supernatants and the corresponding Delta values (virus + LPS) – (LPS only) are reported for each experimental condition in terms of pg/ml*.

## Discussion

On the whole, crucial features of the host/pathogen relationship are still ill-defined in the PRRS model, which bears on the development of effective disease control strategies. In particular, this is true of PRRSV pathogenicity; several virus genome regions may be implied in the regulation of this biological feature, with a major role of those coding for non-structural proteins (25). Yet, a robust, coherent view of the molecular basis of PRRSV virulence is still lacking. PRRSV pathogenicity seems to be crucially affected by genetic features of the virus, genetic features of the host (26), and environmental, infectious, and non-infectious stressors (10). PRRSV circulated for decades in Eastern Europe (23) without causing disease, which appeared after the reunification of Germany in 1990, when PRRSV met the lean type, fast-growing pigs reared in western Europe. These are definitely much more susceptible to PRRSV infection (27). The high level of oxidative stress under resting conditions (28) is probably the Achilles' heel of lean type animals, since oxidative stress underlies a huge amplification of the inflammatory response (29). In this respect, PRRSV shows a notable synergism with LPS in inducing respiratory disease (30), and adverse clinical outcomes of PRRSV infection are correlated with enhanced adaptive immune responses (14). This implies a possible correlation between virus pathogenicity and profile of inflammatory and immune responses to PRRSV.

Our results show that the innate immune responses *in vitro* to PRRSV are affected by virus-specific factors, in agreement with the presence of PRRSV immunotypes (12). This is in line with previous evidence indicating inflammatory cytokines and IL-1beta in particular, as markers of serious clinical outcomes of PRRSV infection. Thus, biologically active IL-1 is dramatically upregulated in bronchoalveolar fluids during PRRSV and LPS-driven respiratory disease (30); serum cytokine levels of IL-1 are upregulated early after PRRSV infection and are correlated with virus persistence (31); exacerbation of disease symptoms after PRRSV challenge is correlated with higher IL-1beta levels in serum (32). The results obtained in this study are apparently at odds with such *in vivo* findings, since only the attenuated reference strain BS114 caused an IL-1beta response to PRRSV in PBMCs, and some pathogenic strains failed to induce any inflammatory response *in vitro*.

Please notice, however, that PBMCs are not competent for PRRSV replication (13). Yet, they are fully reactive to PRRSV as shown by *ex vivo* transcriptome analysis, indicating a major involvement of innate immune response pathways (33). In this scenario, our unexpected results should be interpreted in a global perspective, having in mind the crucial factors underlying macrophage permissiveness for PRRSV replication. Thus, a primary inflammatory response in PBMCs is definitely beneficial to the host because it is associated to non-permissiveness for virus replication of mature macrophages in later phases (34). On the contrary, the lack of a primary inflammatory response and the induction of IL-10 underlie high-titered replication of PRRSV (34). This is also in agreement with circumstantial evidence, showing poor susceptibility to PRRSV of PAM collected from lungs with foci of pneumonia (Amadori, unpublished results). Following high-titered replication in fully susceptible macrophages, PRRSV would be able to induce a major dysregulation of innate immunity (21) and an amplification of the inflammasome response through the small envelope protein E (35), which is counteracted by PRRSV nsp 11 (22). Therefore, the balance between the activities of protein E and nsp 11 might underlie PRRSV-driven regulation of the IL-1beta response. This is in agreement with the observed kinetics of inflammasome response to PRRSV infection, showing rapid induction and early decrease *in vitro* (22). Finally, a persistent IL-10 response to some PRRSV strains might lead to an inflammatory gain under these conditions, as previously shown in a human model (36).

The above conceptual framework is in agreement with the *in vivo* PRRSV–LPS synergistic model, whereby pigs are first inoculated with PRRSV and then inoculated with LPS after a few days (30). This means that an established PRRSV infection sensitizes the lungs for production of proinflammatory cytokines upon exposure to LPS.

Our data confirm that PRRSV strains may induce different patterns of cytokine release (12) and set this tenet into a coherent conceptual framework. In fact, our data indicate that virulence of PRRSV strains is correlated with inhibition to a different extent of the inflammatory response in PBMCs, unable to sustain PRRSV replication. This can be related to a possible immune evasion strategy of virulent PRRSV strains. These would check by unknown mechanisms an early inflammatory response of leukocytes in healthy tissues, to prevent a dramatic restriction of PRRSV replication in later phases. Vice versa, pathogenic PRRSV strains could accrue ongoing inflammatory conditions, as opposed to the attenuated ones. Possibly, the extent of the early inhibition (Table 3) and the extent of the subsequent inflammatory gain after PRRSV replication could both contribute to virulence and adverse clinical outcomes. Needless to say, fully differentiated macrophages are readily available *in vivo* for PRRSV replication, without the need of such an immune evasion strategy.

As for the possible translational repercussions of our work, the described cytokine assays could provide useful indications about the possible risk posed by new PRRSV strains on farm, in addition to previously described *in vitro* assays of pathogenicity based on differentiated macrophages (37, 38). This new approach could be applied to PRRSV strains isolated on farm and shown to significantly differ from the previous ones on the basis of genome sequencing. In this case, the new PRRSV strains could be submitted to the same cytokine assays described in our work to get information about the possible virulence and clinical impact on the local pig population. On the basis of our data (Table 3), no induction of IL-1beta, poor induction of IL-8 (<3–4 ng/ml), and induction of IL-10 to any extent should be taken into account as possible virulence markers of PRRSV.

The observed differences among PRRSV strains highlight the issue of the molecular basis of virulence due to specific features of PRRSV genome. Therefore, *in silico* analysis of PRRSV genome sequences will hopefully highlight the crucial differences between pathogenic and attenuated strains underlying the described biological activities, thus contributing to the development of safe and effective PRRS live-attenuated vaccines.

## Data Availability Statement

The datasets generated for this study can be found in the Gene Bank.

## Ethics Statement

The animal study was reviewed and approved, as shown by the Animal Experiment License issued by the Italian Ministry of Health (n. 631/2017-PR of 7 August 2017).

## Author Contributions

MA supervised the entire study and the manuscript writing. MT contributed to manuscript writing. GF and JR performed cytokine assays. MB and CB performed real-time PCR assays for PRRSV and data analysis thereof. MA and FG performed flow cytometry experiments and data analysis thereof. MT and GA supervised sample collection, storage, and distribution. IB performed PRRSV sequence analyses. All authors contributed to the article and approved the submitted version.

## Conflict of Interest

The authors declare that the research was conducted in the absence of any commercial or financial relationships that could be construed as a potential conflict of interest.

## Abbreviations

PAM: pulmonary alveolar macrophages
PBMCs: peripheral blood mononuclear cells
TCID_50_: tissue culture infectious doses 50%
PRRSV: porcine reproductive and respiratory syndrome virus
SPF: specific pathogen free
RT: reverse transcription
CPE: cytopathic effects
FCB: flow cytometry buffer
MOI: multiplicity of infection
PI: propidium iodide
ATP: adenosine triphosphate
FCS: fetal calf serum
DPI: days postinfection
SD: standard deviation.
