# Supplementary material for: In vitro Cytokine Responses to Virulent PRRS Virus Strains
Source: Front Vet Sci. 2020 Jul 15;7:335. doi: 10.3389/fvets.2020.00335 (PMC7373743; doi:10.3389/fvets.2020.00335)
Supplement: Supplementary file 1 [file Table_1.DOCX]

**Supplementary Table 1**

**Sequences of some PRRSV strains included in the study**

| **PRRSV strains** | **Accession number**  **ORF 5 gene** | **Accession number**  **ORF 7 gene** |
| --- | --- | --- |
| 270433 / 5 | MN617072 | MN617066 |
|  |  |  |
| 271009 / 8 | MN617074 | MN617068 |
|  |  |  |
| 3400 / 2 | MN617075 | MN617069 |
|  |  |  |
|  |  |  |
| 13957 | MN617076 | MN617070 |
|  |  |  |
| 21377 | MN617077 | MN617071 |
|  |  |  |

Gene bank accession numbers are reported for ORF 5 and ORF 7 genes.

The above strains are sometimes reported in a shortened form in the text for convenience.
